# Impact of alternative materials to plasticized PVC infusion tubings on drug sorption and plasticizer release

**DOI:** 10.1038/s41598-019-55113-x

**Published:** 2019-12-12

**Authors:** N. Tokhadze, P. Chennell, L. Bernard, C. Lambert, B. Pereira, B. Mailhot-Jensen, V. Sautou

**Affiliations:** 1 Universite Clermont Auvergne, CHU Clermont-Ferrand, CNRS, SIGMA Clermont, ICCF, F-63000 Clermont-Ferrand, France; 2 Unité De Biostatistiques (Délégation à La Recherche Clinique Et à l’Innovation), CHU de Clermont-Ferrand, 63000 Clermont-Ferrand, France; 3 Universite Clermont Auvergne, CNRS, SIGMA Clermont, ICCF, F-63000 Clermont-Ferrand, France

**Keywords:** Therapeutics, Drug delivery

## Abstract

Medical tubings in plasticized polyvinylchloride (PVC) are widely used for the infusion of medications but are known in some cases to cause content-container interactions (drug sorption and plasticizer release). The aim of this study was to assess interactions between drugs and five alternative materials to a reference plasticized PVC intravenous (IV) infusion tubing: three were PVC coextruded with polyethylene (PE), polyurethane (PU) or a thermoplastic elastomer (Styrene-EthyleneButadiene-Styrene (SEBS)) and two were SEBS or thermoplastic olefin (TPO) monolayer tubings. Diazepam and insulin were chosen as respective reference of absorption and adsorption while paracetamol acted as a negative control. The concentration of each drug was quantified with liquid chromatography to evaluate a potential loss after a static contact condition and simulated infusion at 1 mL/h and 10 mL/h dynamic condition by an electric syringe pump. A characterization of each material’s surface was performed by Fourier transform infrared spectroscopy in attenuated total reflection mode (ATR-FTIR) and by measurement of surface zeta potential. Plasticizer release was quantified by gas chromatography coupled with mass spectrometry (GC-MS). For all tubings except PVC/PU, no loss of paracetamol was observed in any condition. Diazepam sorption appeared to be less important with PVC/PE, PVC/SEBS, SEBS and TPO tubings than with PVC, but was more important when using PVC/PU tubings. PVC tubings induced the least loss of insulin amongst all the studied materials. Surface analysis by ATR-FTIR highlighted the presence of a plasticizer (that could be attributed to Tris (2-Ethylhexyl) Trimellitate (TOTM)) in the coextruded SEBS layer of PVC/SEBS, which could have influenced drug sorption, probably as a consequence of a migration from the PVC layer. Coextruded PVC/SEBS and PVC/PE presented the lowest zeta potential of all studied materials with respective values of −39 mV and −36 mV and were related to the highest sorption of insulin while PVC/PU with the highest zeta potential (about −9 mV) presented the highest absorption of diazepam. Coextruded layered materials appeared to have a lower plasticizer release than PVC alone. As a conclusion, PVC/PE and thermoplastic elastomers alone or coextruded with PVC could be interesting alternatives to PVC tubings with regards to sorption phenomena and plasticizer release.

## Introduction

Because of its good mechanical properties combined with a low cost of fabrication, PVC has been widely used for the manufacture of IV tubings. Yet this material is not completely inert when infusing drug solutions. It can affect drug solutions by releasing compounds into the infusate or by retaining the drug (sorption) thus potentially affecting infusion safety and effectiveness. Sorption phenomena can cause a loss of active pharmaceutical ingredient (API)^1,2^ or protective excipients^3^ and are mediated by different physicochemical parameters/properties^4–6^ that are still incorrectly evaluated. The phenomena can be detailed in two steps: adsorption then absorption. Adsorption is the result of a weak interaction between a compound in solution and a surface. This phenomenon is fast and reversible. Absorption corresponds to the diffusion of a molecule inside the material. It is slower and comes after adsorption. As Peterfreund *et al*. reported^7^, sorption related loss of drug is underappreciated. This issue was reported with PVC bags and tubings from the 80’s^4^ with different drugs such as diazepam^8,9^, amiodarone^10^, isosorbide dinitrate^11^, insulin^12,13^. More recently, many studies also highlighted losses of drug during administration with PVC but also with non-PVC based catheters or IV tubings^2,14–17^. Even though the sorption issue has been known for a long time, the mechanisms involved during this phenomenon are not completely elucidated.

Moreover when using PVC based IV tubings, plasticizer leaching is a major concern. Plasticizers are compounds added to the PVC to make it more flexible, and these products can be released into the infused medication and then in the bloodstream. Until about ten years ago, the most used plasticizer was the Di(ethylhexyl)phthalate (DEHP) which was regulated because of its toxicity. Alternative plasticizers like Diisononyl Phtalate (DINP), Di-(2-Ethylhexyl) Phtalate (DEHT), 1,2-cyclohexane dicarboxylic acid diisononyl ester (DINCH) or Tris(2-Ethylhexyl) trimellitate (TOTM) were then used in the manufacturing of medical devices^18^, yet all those alternatives plasticizers can potentially migrate from the PVC matrix. Recently, our research team has shown that the addition of a coextruded inner layer of polyethylene (PE) in PVC infusion tubings appears to reduce plasticizer release^19^, but the effect of other coextruded materials like polyurethane on plasticizer release has not yet been studied. In addition to their potential toxic effects, plasticizers have also been shown to have an influence on drug sorption^20,21^.

Factors affecting sorption are related to the physicochemical properties of the drug itself (lipophilicity, pKa, isoelectric point, steric hindrance, concentration), but are also related to the excipient composition, infusing parameters (flowrate, medical devices length) and the physicochemical properties of the polymer constituting the IV tubings. Identifying and evaluating material related factors appears as a challenging way to provide information to better understand drug sorption, and could help identify at-risk situations and select the best material for IV-tubings, and thus improve the control of the administered dose to the patient.

The aim of this study was to assess the sorption in conditions simulating the clinical use of three drugs with PVC and 5 alternative materials (co-extruded with an inner layer of polyethylene (PE), polyurethane (PU) and styrene-ethylenebutadiene-styrene block copolymer (SEBS), bulk SEBS and a bulk thermoplastic olefin (TPO)). For each material, the influence of surface physicochemical properties in the sorption process was investigated. The three molecules that were studied were diazepam which is an API that has been known for many years to absorb into medical tubings^20,22^, insulin which is subject to adsorption only^13,17^ and paracetamol which was used as a negative control (not reported to be sensitive to sorption phenomena). In order to provide additional information about mechanisms involved in the sorption process, the inner surface of each IV tubings was also characterized. The majority plasticizer migration potential was also evaluated to assess the impact of the coextruded layer on plasticizer migration.

## Materials and Methods

### Materials

#### Medical devices

The tubings used are described in Table 1.

**Table 1:** Description of IV-tubings (PVC: Polyvinylchloride; PE: Polyethylene; PU: Polyurethane; SEBS: Styrene-Ethylenebutadiene-Styrene; TPO: Thermoplastic olefin)

Inner surface material	PVC	PE	PU	SEBS	SEBS*	TPO*
Coextrusion with	/	PVC	PVC	PVC	/	/
Reference Batch/Grade Manufacturer	PN10318-1 118D06-TE Cair LGL, France	RPB5320 16F27 Cair LGL, France	PY2301NCM 17F06-T Cair LGL, France	PT050117 17B03-TE Cair LGL, France	Cawiton tubing grade PR13816 Wittenburg, The Netherlands	Cawiton tubing grade PR13997 Wittenburg, The Netherlands
Length (cm)	200	200	10	150	200	200
Internal/external Diameter (mm)	2.5/4.1	2.5/4.1	2.5/4.1	2.5/4.1	2.5/4.1	2.5/4.1
Inner Surface Area (cm²)	157.08	157.08	7.85	117.81	157.08	157.08

^*^SEBS and TPO tubings were of medical grade but not obtained from commercialized extension sets.

For PVC, PVC/PE, PVC/PU and PVC/SEBS, the PVC matrix was plasticized with plasticizer tris(2-ethylhexyl) trimellitate (TOTM) (manufacturer information).

50 mL Luer Lock polypropylene syringes (BD Plastipak®, reference 300865 (Becton Dickinson, France), batches 1712226 and 1701265 P, expiring respectively 11/2022 and 12/2021)) were also used as storage container during infusion simulation.

#### Medications

The following marketed medications were used:

- VALIUM® (Diazepam) 10 mg/2 mL (Roche, Rosny-sous-Bois, France; batch F1126F01, expiring 09/2020).
- NOVORAPID® (Insulin aspart) 100 UI/mL (Novo Nordisk, Courbevoie, France; batch HS65E14, expiring 01/2020). Insulin aspart will henceforth be referred to as insulin.
- Paracetamol B BRAUN® (paracetamol) 10 mg/mL (B. Braun, Saint Cloud, France; batch 18105452, expiring 02/2020 and 18141450, expiring 03/2020).

Physicochemical properties, and Van der Waals volume, of each API were obtained from Chemicalize®^23^. For diazepam, insulin and paracetamol, partition coefficient (logP) were respectively of 3.08, −18.85 and 0.91 and Van der Waals volume were respectively of 242.85Å^3^, 3123.51Å^3^ and 138.08Å^3^.

#### Reagents

The following reagents were used for chromatographic separation: acetonitrile (ACN) 99% purity (Fisher Chemical, United Kingdom); methanol 99% purity (Fisher Chemical, United Kingdom); formic acid 98% purity (Fluka, Germany), trifluoroacetic acid (TFA) (Sigma-Aldrich, Germany); monobasic potassium phosphate (Sigma-Aldrich, Germany). All reagents were of certified HPLC grade.

### Methods

#### Study design

Sorption phenomena were evaluated by quantification of the API after static and dynamic contact between all medications and IV tubings. Static condition simulated a worst-case scenario in which drugs and materials were put in contact during 96 hours without renewal of the solution. Dynamic contact aimed to simulate an 8 hours infusion at two different flowrates.

Each analysis was performed in triplicate. All devices and equipment used for the preparation of the drugs, the conditioning and the sample withdrawal were chosen so as to avoid any kind of content-container interactions, and the physicochemical inertia relative to the sorption phenomena was preliminarily checked.

#### Sample preparation

The studied solutions were prepared as follow, by dilution from the commercial medications and in accordance with the summary of product recommendations:

- Paracetamol: diluted to 1 mg/mL in a 0.9% sodium chloride solution Versylene® (Fresenius Kabi, France).
- Diazepam: diluted to 0.2 mg/mL in a 5% glucose solution B. Braun® solution (B.Braun, Germany)
- Insulin: diluted to 0.1 UI/mL in a 0.9% sodium chloride solution Versylene® (Fresenius Kabi, France.

The final concentrations were chosen to be representative of clinically used concentrations.

#### Static study

For each condition, the IV-tubings were filled with each drug at the studied concentration. After filling, the tubings were clamped and stored in standardized conditions in a validated climate chamber (Binder, model KBF240, GmbH Tuttligen, Germany) at 25 °C ± 2 °C and 60% humidity, in the dark.

For each sample, in the syringe before tubing filling (initial time Ti), then from the tubings right after filling and purge (T0) and after 24 and 96 hours of contact (further referred to as T0, T24 and T96) a visual control and API quantification was performed. For each analytical time, three different tubings (n = 3) were used: the tubings were fully emptied and discarded after analysis. Thus, for the static study a total of 9 units were used.

#### Dynamic study

**Figure 1:**
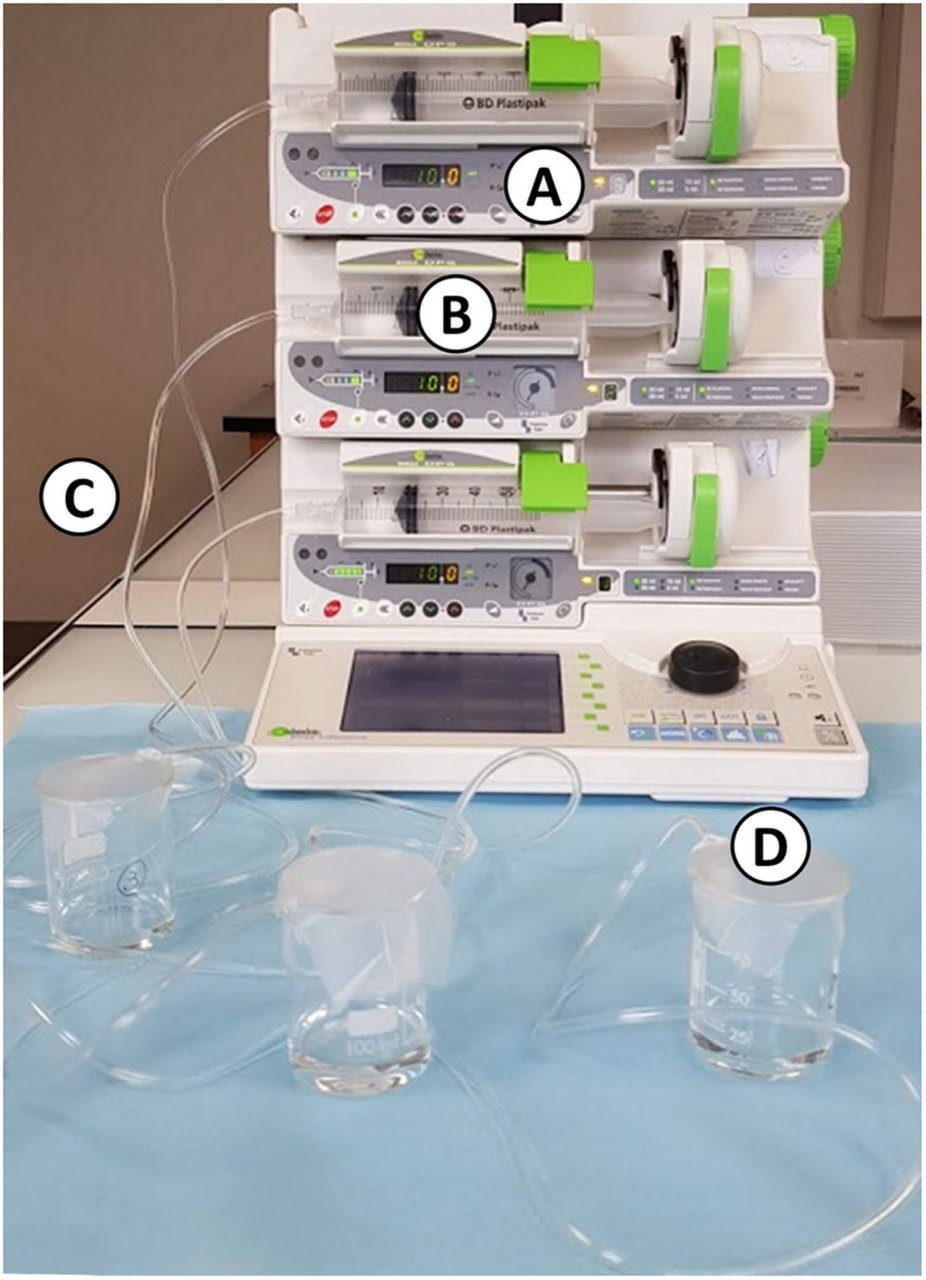
Picture of the experimental setup in dynamic condition (**A**) electric syringe pump; (**B**) 50 mL syringe; (**C**) infusion tubing; (**D**) withdrawing site at the tip of the tube).

A simulation of an IV infusion using an electric syringe pump (Orchestra® DPS modules, Fresenius, France) was performed at two different flowrates: 1 mL/h and 10 mL/h, which are flowrates commonly used for IV drug infusion. The experimental setup is presented in Fig. 1.

For each condition, a sample of the drug solution was collected from the tip of the syringe before contact with the tubing (Ti), then at T0 at the tip of the tubing, after purging. Other samples were collected at the tip of the tubing without stopping the infusion, after 1, 2, 4 and 8 hours of simulated infusion. An approximate volume of 150 µL was collected for each analysis time (minimum volume needed to perform the quantitative analysis) and thus the sampling time was dependent of the flowrate (about 1 min and 10 min respectively for the 10 mL/h and 1 mL/h condition). Visual control and API quantification were performed on the samples.

### Analyses

After preparation, visual examination of diluted solutions was performed and pH was measured. For all analytical time, API was quantified after separation by HPLC.

Evaluation of the physicochemical properties of the tubings inner surface was performed by ATR-FTIR spectroscopy and zeta potential measurement.

For PVC or coextruded PVC tubings, the released plasticizer was identified and its migration quantified.

#### Visual examination and pH measurement

Each collected sample was visually controlled and compared to a freshly prepared sample. Immediately after preparation pH was measured using a SevenMultiTM pH-meter with an InLab MicroPro glass electrode (Mettler-Toledo, Viroflay, France).

#### API quantification

At each analytical time, API was quantified using one of the following high-pressure liquid chromatography systems and integrated data treatment software:

- AS4150 autosampler, PU4180 pump, CO-4061 oven, and MD-2018 diode array detector (Jasco, Bouguenay, France)
- LC-2010HT compact system (Shimadzu, France).

The in-house chromatography methods used for the quantification of the API are presented in Table 2.

**Table 2:** Chromatography methods used for quantification of paracetamol, diazepam and insulin. TFA: trifluoroacetic acid.

API	Chromatography column	Mobile Phase	Mode	Flow rate (mL/min)	Oven Temperature (°C)	Detection wavelength (nm)	Injection volume (µL)
Paracetamol	Gemini C18 3 µm, 150 × 4,6 mm Precolumn Gemini C18 5 µm, 0,04 × 0,03 cm (Phenomenex, France)	Phase A: Water adjusted to pH 2.75 with formic acid 98% Phase B: Acetonitrile	Gradient (% Phase A): 0–3 min: 90% 3–7 min: 90% to 40% 7–10 min: 40%	1.3	40	243	10
Diazepam	Nucleodur C18 HTEC 5 µm, 125 × 4,6 mm (Macherey-Nagel, France)	v/v/v 22% Acetonitrile 34% Methanol 44% phosphate buffer (3.4 g/L, pH = 5.0)	isocratic	1.0	30	254	20
Insulin	Nucleodur C18 ec 5 µm, 250 × 4,6 mm (Macherey Nagel, France)	Phase A: TFA/water 0,1% (v/v) Phase B: TFA/acetonitrile 0,08% (v/v)	Gradient (% Phase A): 0–15 min: 80% to 35% 15–17 min: 35% 17–20 min: 35% to 80%	1.3	25	280	20

The linearity of the method was verified through the analysis of 3 independent calibration ranges performed on solutions for each API on 3 different days. The mean accuracy, the repeatability and the intermediate precision were calculated through repeated quantitative analysis of 6 independent solutions, repeated on 3 different days. For this study, the limit of detection was validated as the lowest point of the calibration curve, except for insulin for which an optimized quantification limit was researched.

All samples were diluted to within theoretical calibration curve range, and if beneath quantification limit the samples were reanalyzed after adapting the dilution.

#### Expression of the results of API quantification

For all three tested API, the results were expressed as the percentage of the initial concentration (measured at Ti). Error bars expressed the 95% confidence interval of the mean value.

In order to make the results of API quantification comparable from one molecule to one another and from one tubing to one another, the percentage of the initial concentration was divided by the surface contact area of the tubings. Sorption rates were calculated with Eq. 1, and expressed as a percentage of sorption per square centimeter of tubing.

*Equation* *1**: Calculation of the sorption rate standardized by area of contact between drug solution and tubings inner material*

1 $$Sorption=\frac{Ci-Cf}{Ci}\,\times \frac{1}{S}\times 100$$

*C*_*i*_: initial concentration (mM)

*C*_*f*_: final concentration (mM)

*S*: inner surface area (cm^2^)

#### Statistical analysis

All statistical analyses were performed using Stata statistical software (version 13, StataCorp, College Station, US). The tests were two-sided, with a type I error set at 5%. Continuous parameters were expressed as mean ± standard-error of mean (SEM) according to statistical distribution (assumption of normality studied using Shapiro-Wilk’s test).

To study longitudinal evolution, correlated repeated data were analyzed using linear mixed models. This approach seems more relevant rather than usual statistical tests because assumption concerning independence of data is not met. The (fixed) effects group, time-point evaluation and their interactions time x flow rate were studied. The normality of residuals from these models has been studied using the Shapiro-Wilk test. When appropriate, a logarithmic transformation was proposed to achieve the normality of dependent data. A Sidak’s correction of the type I error was applied to take into account multiple comparisons. Finally, Bayesian Information Criterion (BIC) was estimated to determine the most appropriate model, notably concerning the covariance structure for the random-effects due to repeated measures across the time and consequently to the autocorrelation.

Concerning comparisons involving non-repeated data, the quantitative variables were compared between independent groups by ANOVA or Student t-test. The assumptions of ANOVA and t-test were evaluated. More precisely, the homoscedasticity was analyzed using the Bartlett test. Furthermore, when appropriate, post-hoc tests were performed to take into account multiple comparisons (Tukey-Kramer post ANOVA and Dunn after Kruskal-Wallis). Hedges’ g effect sizes (ES)^24^, calculated as presented in Equation 2, and 95% confidence intervals (CI) were calculated at T8 between PVC and each alternative tubings. Effect size can be interpreted according to Cohen’s recommendations^25^. A negative effect size is indicative of the material inducing a higher sorption rate than the PVC reference material, whilst a positive effect size was indicative of a lower tendency to promote sorption of the tested material compared to PVC tubings. Forest-plots were used to represent graphically these results.

*Equation 2: Hedge’s effect sizes calculation*

$$ES=\,\frac{{m}_{1}-{m}_{2}}{S{D}_{pooled}}=\frac{{m}_{1}-{m}_{2}}{\sqrt{\frac{({n}_{1}-1){s}_{1}^{2}+({n}_{2}-1){s}_{2}^{2}}{{n}_{1}+{n}_{2}-2}}}$$

*m*_1_ and *m*_2_: mean at T8 for PVC (m_1_) and alternative tubing (m_2_)

n_1_ and n_2_: sample sizes

*s*_1_ and *s*_2_: standard deviation

#### FTIR

ATR-FTIR spectra of the inner surface of each tubing were acquired with a spectrum 100 spectrometer (PerkinElmer) equipped with an ATR diamond crystal. All spectra were acquired from 3500 to 650 cm^−1^, using 16 scans with a 2 cm^−1^ resolution.

#### Surface Zeta potential measurements

In contact with an aqueous solution, a solid surface assumes a surface charge. The Zeta potential (or electrokinetic potential) describes the charging behavior at interfaces. Surface Zeta potential is representative of the electric charge at the shear plane between the diffuse layer and the immobile layer of a material. The surface Zeta potential of the inner surface (before any drug administration) of all tested IV-tubings was assessed by measuring the streaming potential with a Surpass 3 (Anton Paar, France) equipped with a tubing cell analysis system, in a 1 mmol/L potassium chloride solution at pH 5 before analysis in order to standardize the conditions.

#### Plasticizer quantification

The amount of the plasticizer in the PVC matrix (for the PVC containing tubings) was quantified by gas chromatography coupled to a mass spectrometer (GC-MS) with the chromatographic method and extraction process developed by Bourdeaux *et al*.^26^. The plasticizer migration was assessed following the model published by Bernard *et al*.^27^.

## Results

### Validation of API quantification

The analytical method validation data of each API is presented in Table 3.

**Table 3:** Analytical method validation data. CV: coefficient of variation.

API	Calibration curve	Slope of the regression line	Intercept of the regression line	Coefficient of determination (r²)	Mean repeatability CV (%)	Mean intermediate precision (%)	Relative mean trueness bias (%)
Paracetamol	60–140 µg/mL	28742342.0	−40415.1	0.996	0.50	4.70	0.58
Diazepam	1–40 µg/mL	64074.1	−13506.0	0.986	2.80	3.90	0.27
Insulin	0.06–0.14 U/mL	27308.3	−1991.1	0.984	6.40	6.40	1.04

The mean coefficients of variation are under 5%, and mean coefficients of determination above 0.99 for paracetamol and diazepam. A slightly more important variability was noted for insulin. The methods can therefore be considered as linear, accurate, true and repeatable for the tested conditions.

The limit of quantification of insulin was fixed at 0.03 UI/mL, limit at which the mean coefficients of repeatability, intermediate precision and relative trueness bias were of 4.08%, 8.31% and 12.27%, respectively. Insulin concentrations between 0.03 and 0.06 UI/mL were taken into account for their indicative value only.

### API quantification

#### Paracetamol

**Figure 2:**
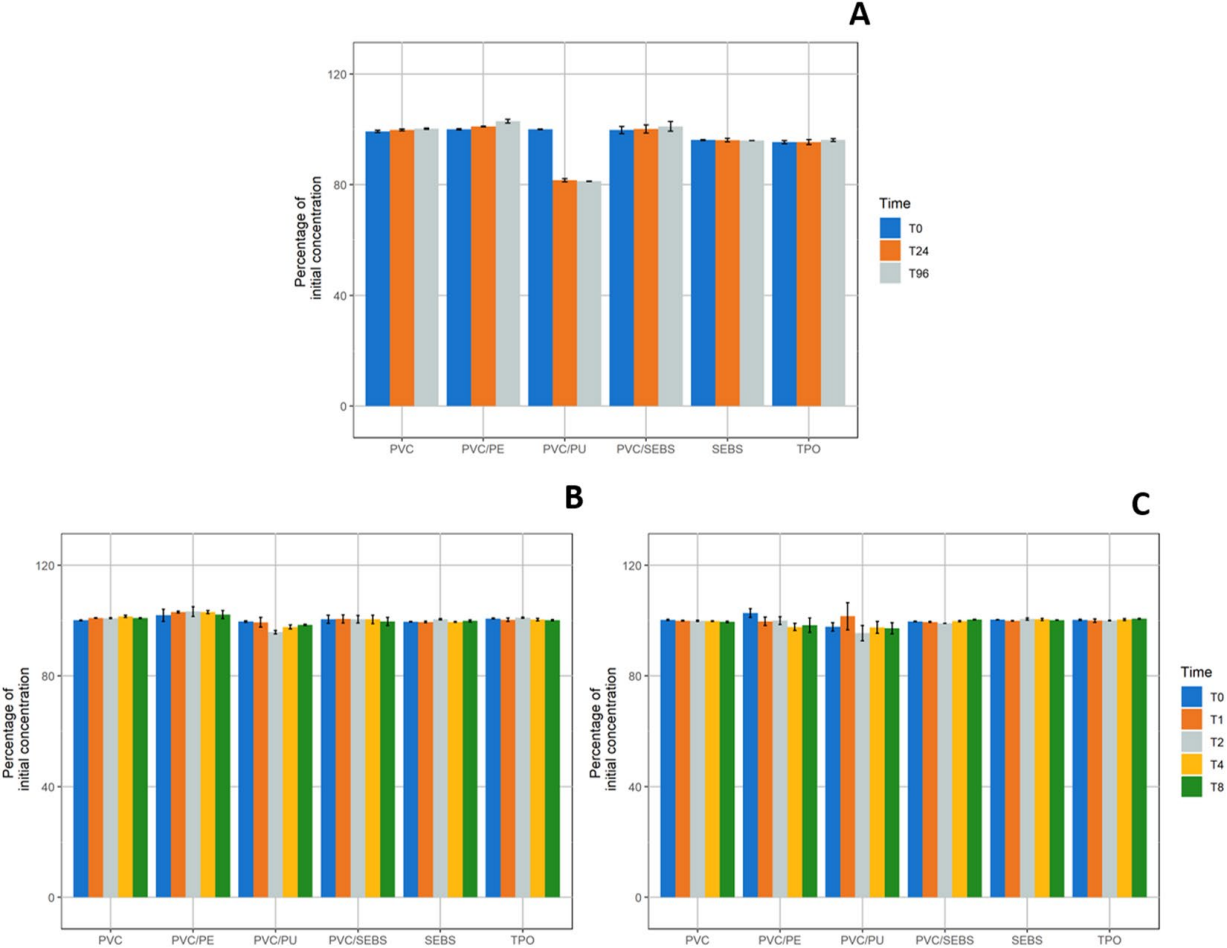
Evolution of paracetamol concentrations compared to initial concentration in static condition (**A**); 1 mL/h dynamic (**B**) and 10 mL/h dynamic condition (**C**) for every studied tubing (n = 3, mean ± standard error of mean). (PVC: Polyvinylchloride; PE: Polyethylene; PU: Polyurethane; SEBS: Styrene-Ethylenebutadiene-Styrene; TPO: Thermoplastic olefin).

At the initial time of every studied condition, paracetamol concentrations in solution were comprised between 0.913 mg/mL and 1.045 mg/mL and pH was of 5.30. Paracetamol percentages of initial concentrations measured after static or dynamic contract with IV-tubings are presented in Fig. 2.

Paracetamol concentrations did not vary by more than 10% of Ti concentration for every condition except for PVC/PU IV-tubings in static condition for which paracetamol concentration decreased to 81.61% ± 0.57% at T24 and 81.23% ± 0.08% at T96.

Paracetamol sorption relative to contact surface area (in cm²) showed a significant increase with time (p < 0.001) only for PVC/PU samples, respectively +2.39 ± 0.01%/cm² at T96 in static condition and +0.20 ± 0.06%/cm² at T8 in 1 mL/h dynamic condition (see details in Supplementary Data, Fig. A).

#### Diazepam

**Figure 3:**
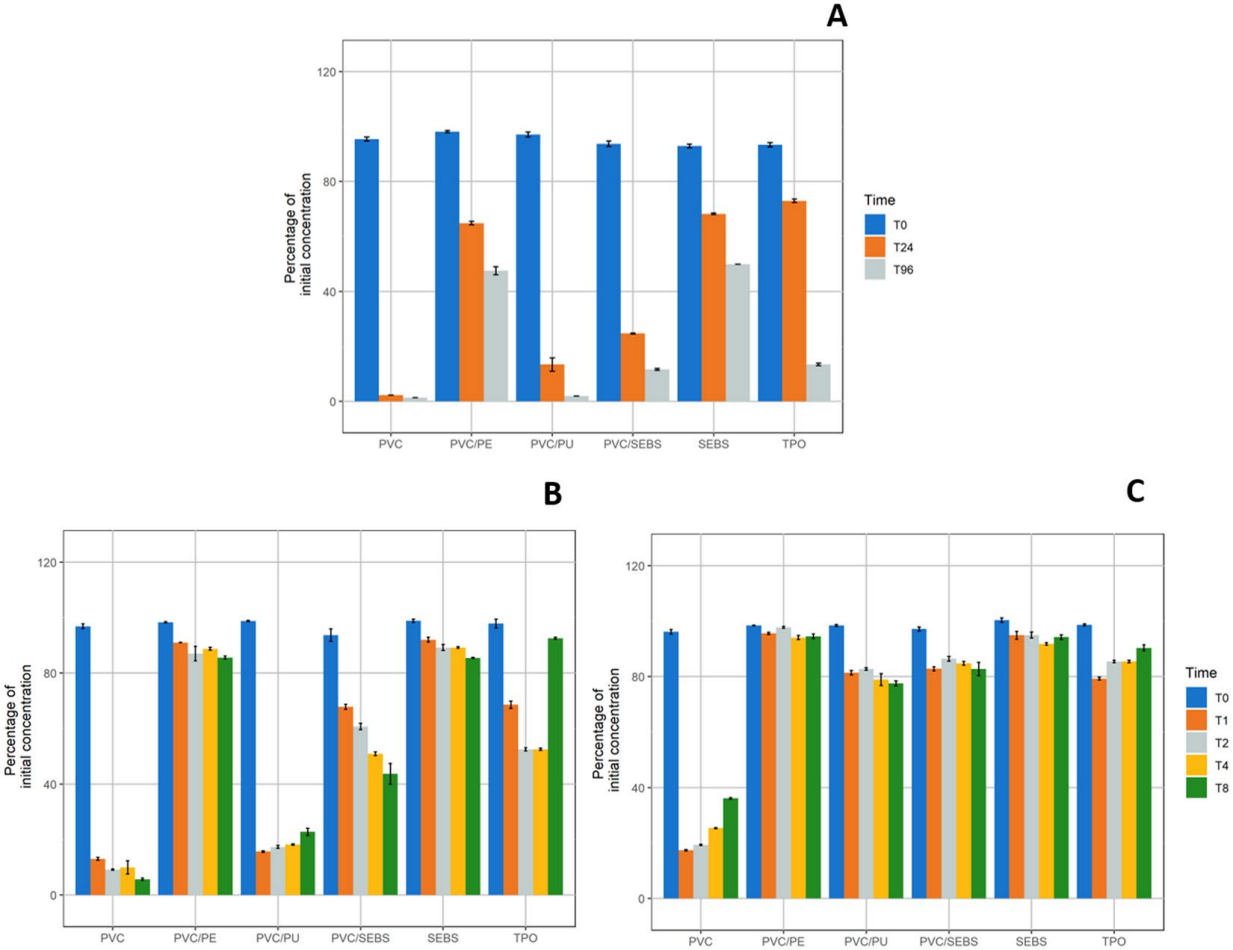
Evolution of diazepam concentrations compared to initial concentration in static condition (**A**); 1 mL/h dynamic (**B**) and 10 mL/h dynamic condition (**C**) for every studied tubing (n = 3, mean ± standard error of mean). (PVC: Polyvinylchloride; PE: Polyethylene; PU: Polyurethane; SEBS: Styrene-Ethylenebutadiene-Styrene; TPO: Thermoplastic olefin).

For all materials and flowrate conditions, diazepam concentrations in solution before infusion were comprised between 0.18 mg/mL and 0.21 mg/mL and pH was of 5.38. The variation from diazepam Ti concentrations is shown in Fig. 3.

In static condition, diazepam concentrations decreased for every tested material after 24 hours contact (T24) and at T96, with most important losses noticed for PVC and PVC/PU (residual diazepam percentages being of 1.29% ± 0.00% and 1.88% ± 0.03% respectively for PVC and PVC/PU tubings after 96 hours of contact). The materials inducing the least loss were PVC/PE and SEBS (residual diazepam concentrations of respectively 47.52% ± 1.45% and 49.86% ± 0.02% at T96).

During the 1 mL/h dynamic condition, a decrease of diazepam concentrations was observed right from T1 for every material samples, with concentrations ranging from 13% to 92% of initial concentration, function of the material. In correlation with the result of static condition, groups with different behaviors were observed. PVC and PVC/PU tubings induced the most important loss right from T1 (residual concentrations of about 15%) then remained stable throughout the rest of the study. For PVC/PE and SEBS tubings, the remaining concentrations remained over 85% for all analytical times. With PVC/SEBS and TPO tubings, the loss of diazepam was progressive until T4 (about 50% of initial concentration), but at T8 the decrease continued to up to 40% with PVC/SEBS tubings, while diazepam concentration in TPO tubings increased to 92%.

During the 10 mL/h dynamic condition, diazepam concentrations decreased less, with concentrations comprised between 77% and 97% for each tested material, except for PVC tubings where a minimum of 17.41% ± 0.21% was reached at T1, before raising up again to 36.10% ± 0.27% at T8.

For all analytical time, conditions and materials, diazepam sorption/cm² was statistically significantly higher (p < 0.001) when compared to sorption at T0 (Supplementary Data, Fig. B), indicating that observed decrease in concentrations is significant.

#### Insulin

**Figure 4:**
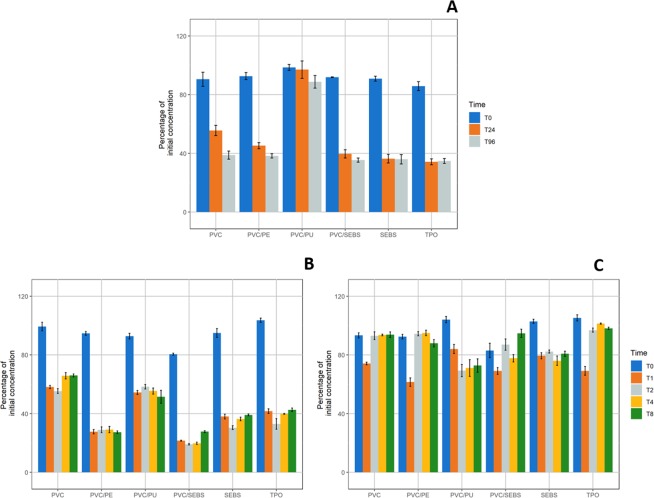
Evolution of insulin concentration compared to initial concentration in static condition (**A**); 1 mL/h dynamic (**B**) and 10 mL/h dynamic condition (**C**) for every studied tubings (n = 3, mean ± standard error of mean). (PVC: Polyvinylchloride; PE: Polyethylene; PU: Polyurethane; SEBS: Styrene-Ethylenebutadiene-Styrene; TPO: Thermoplastic olefin).

Initial concentrations of all insulin samples were comprised between 0.083 and 0.115 IU/mL and pH was of 6.44. As shown in Fig. 4, insulin concentrations varied differently between static and dynamic condition.

In static condition, insulin concentrations decreased to around 40% at T24 then remained almost stable for all tested materials except for PVC/PU tubings where they remained almost stable from T0 to T96.

During the 1 mL/h dynamic condition, insulin concentrations decreased at T1 then remained stable for the other analytical times for every tubing. However, three groups with different behaviors were observed: the loss was most important for PVC/PE and PVC/SEBS tubings (about 75% loss), SEBS and TPO had an intermediate behavior (about 60% decrease), while PVC and PVC/PU were the tubings that induced the least loss of insulin, as about 50% of initial concentration remained.

With a 10 mL/h flowrate, two behaviors were observed. On one side, PVC, PVC/PE, PVC/SEBS and TPO tubings presented a loss of insulin at T1, but concentrations then returned to about 100% of initial values from T2 to T8. On the other side, contact with PVC/PU and SEBS samples induced a decrease of about 20% of initial concentration from T1 to T8.

For all any analytical times, insulin sorption relative to contact surface area (sorption/cm2) in static condition was statistically significantly increased (p < 0.001) when compared to T0 for all tubings except for PVC/PU (see details in Supplementary Data, Fig. C). In the 1 mL/h dynamic condition, sorption/cm² was significantly (p < 0.001) increased for all analytical times when compared to T0 for all IV tubings. In the 10 mL/h dynamic condition, insulin sorption/cm² at T1 was different from T0 (p < 0.001), but not at any other analytical time for PVC, PVC/PE, PVC/SEBS and PVC/TPO tubings. For other tubings, insulin sorption/cm² was significantly different from T0 at all analytical time (except for PVC/SEBS at T4).

### Effect size

**Figure 5:**
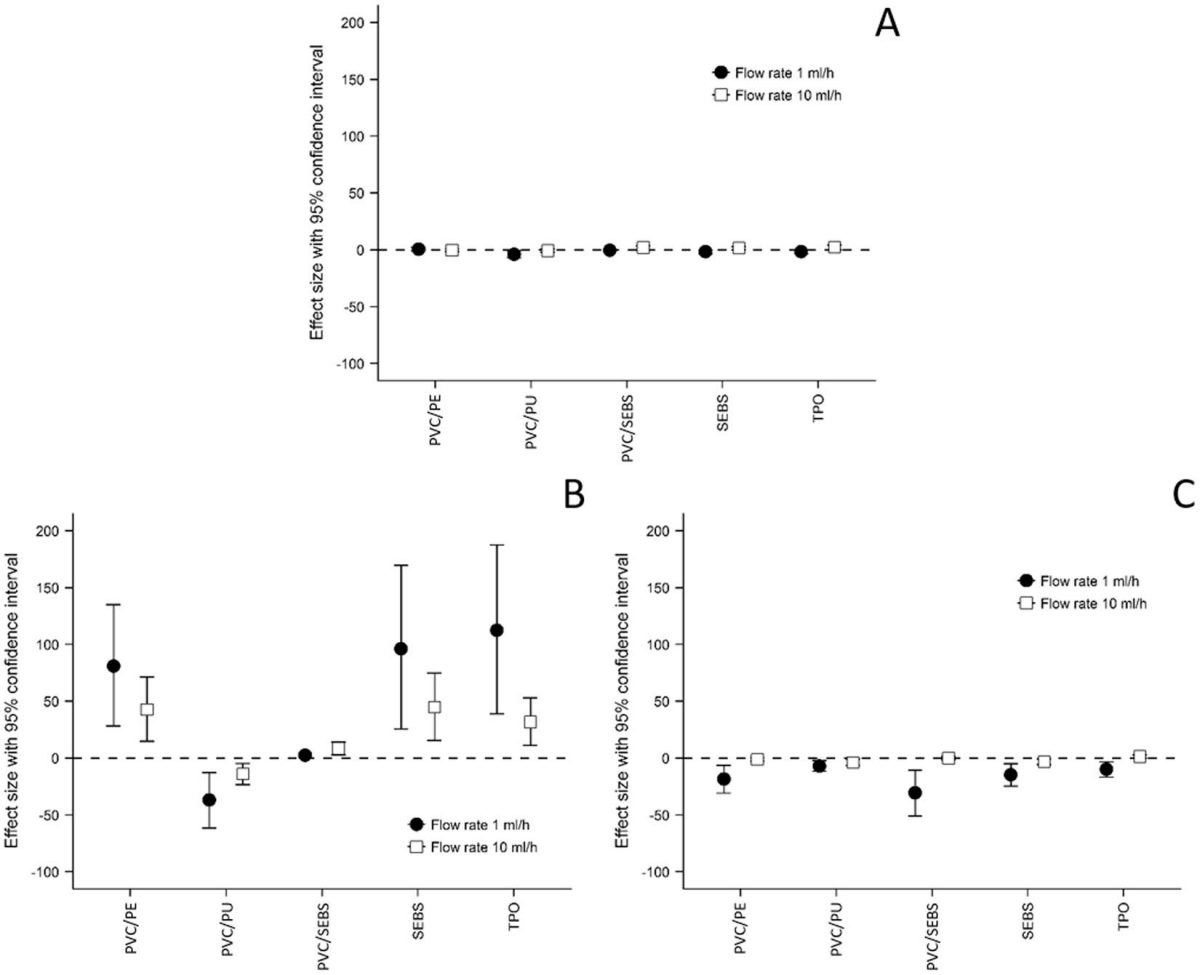
Effect size of the comparison of each material to PVC after an 8 h infusion at 1 ml/h and 10 ml/h. (**A**) Paracetamol; (**B**) Diazepam; (**C**) Insulin (mean ± confidence interval of 95%). (PVC: Polyvinylchloride; PE: Polyethylene; PU: Polyurethane; SEBS: Styrene-Ethylenebutadiene-Styrene; TPO: Thermoplastic olefin).

PVC was chosen as the reference tubing material and all the other tubings were compared to this reference at the final analytical time (T8). A comparison of effect sizes is presented in Fig. 5.

For all molecules, absolute value of effect size was reduced when the flow rate was increased. During paracetamol contact, only the PVC/PU tubings at a flow rate of 1 mL/h appeared to be significantly different from 0 thus different from PVC since it was comprised between −6.80 and −1.00. During diazepam infusion, only the PVC/PU samples had a negative effect size (for both 1 mL/h and 10 mL/h conditions). PVC/SEBS tubings had an ES comprised between 0.37 and 4.58, which was significantly higher than PVC but also significantly lower than PVC/PE, SEBS and TPO tubings. These last three tubings had a positive effect (less sorption) but not significantly different from one another. During insulin infusion, all materials appeared to have a negative effect size (exhibiting higher sorption levels) at a flow rate of 1 mL/h, and were significantly different from PVC.

### FTIR

**Figure 6:**
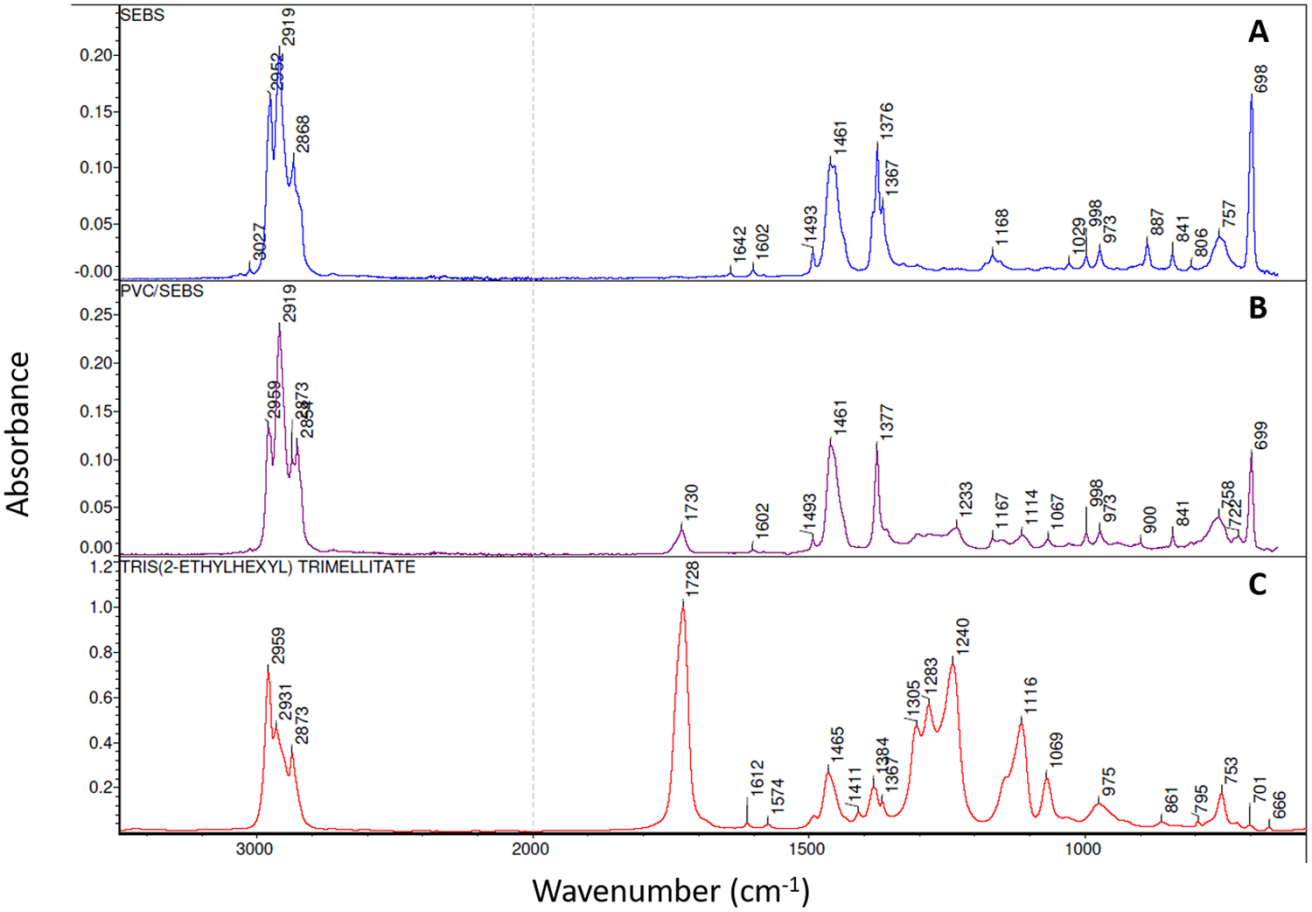
FTIR spectra of the inner surface before infusion of SEBS (**A**) and PVC/SEBS (**B**) tubings compared to TOTM FTIR spectrum (**C**) (PVC: Polyvinylchloride; SEBS: Styrene-Ethylenebutadiene-Styrene).

The inner surface of each studied extension set was analyzed by FTIR spectroscopy. SEBS and PVC/SEBS spectra are presented in Fig. 6. Bulk SEBS and coextruded SEBS spectra showed many similarities, indicating close chemical composition. However, coextruded SEBS presented additional bands (1730, 1304, 1282 1233 and 1114 cm^−1^), that could be attributed to TOTM (see TOTM reference spectra presented Fig. 6(C). Based on this result, it is possible that TOTM is present in the analyzed surface. PVC, PU, PE and TPO spectra are presented in Supplementary Data (Fig. D) and conformed to reference spectra.

### Surface zeta potential

The inner surface charge of every tubing was estimated by measuring its surface zeta potential (Table 4).

**Table 4:** zeta potential (mV) of the inner surface of every tubings at pH = 5.0 (PVC: Polyvinylchloride; PE: Polyethylene; PU: Polyurethane; SEBS: Styrene-Ethylenebutadiene-Styrene; TPO: Thermoplastic olefin).

class	PVC	PVC/PE	PVC/PU	PVC/SEBS	SEBS	TPO
	thermoplastic	thermoplastic	thermoplastic	thermoplastic elastomer	thermoplastic elastomer	thermoplastic elastomer
co-extruded	no	PVC	PVC	PVC	no	no
pH	5.06	4.98	5.06	5.04	5.00	5.04
zeta potential (mv)	−27.4	−37.0	−9.5	−39.6	−33.1	−30.1

Only the PVC/PU tubing presented a surface zeta potential different from the other tubings (−9.5 mV). All the other five tubings had surface zeta potential ranging from −27.4 (PVC) to −39.6 mV (PVC/SEBS).

### Plasticizer migration

The majority plasticizer present in the PVC and PVC coextruded with PE, PU and SEBS was TOTM. As shown in Table 5, TOTM was quantified in the PVC tubings and coextruded PVC and its migration was estimated. Percentages of TOTM in plasticized PVC tubings were not statistically different from those in the external PVC layer of coextruded tubings. However, the release of TOTM was significantly lower with coextruded tubings than with PVC alone with an < 0.01 p-value (T-test) for all coextruded materials. TOTM release from PVC/PU tubings was more important than from PVC/PE tubings (T-test, p < 0.01) and from PVC/SEBS tubings (T-test, p < 0.01).

**Table 5:** Quantification of plasticizer (Tris(2-ethylheyl) trimellitate: TOTM) in Polyvinyl Chloride (PVC) and PVC coextruded tubings and plasticizer migration assay (n = 3, mean ± standard error of mean).

Infusion tubings	Percentage of TOTM in PVC (% w/w)	Quantity of TOTM released per cm² of tubing after 24 h (µg/cm²)
PVC	48.04 ± 7.39	11.45 ± 0.09
PVC/PE	50.35 ± 1.92	3.17 ± 0.01
PVC/PU	46.14 ± 1.90	7.57 ± 0.43
PVC/SEBS	42.41 ± 2.69	5.39 ± 0.15

## Discussion

Our study presents new results that bring complementary information about the interactions between medications and new alternative to PVC polymer materials used for drug infusions in conditions simulating clinical administration. The materials interacted differently with active ingredients depending on the characteristics of the drugs and the flow rate. The overall result of effect size calculation based on the comparison of sorption rates at T8 between PVC and alternative tubings (taking into account surface contact area of the tubings) highlighted that the PVC/PU IV tubings were more prone to drug sorption than PVC with all tested molecules while PE and thermoplastic elastomers (PVC/SEBS, SEBS and TPO) had a better behavior than PVC when in contact with diazepam. An adsorption phenomenon was observed for all IV tubings when in contact with insulin, yet differences between each material were less important than for other tested drugs. This study also highlights that the analysis of material surface physicochemical properties by zeta potential measurement was an innovative and interesting approach for the characterization of medication mediated content-container interaction and brought information about factors involved in drug sorption.

Three medications were chosen as models because of their different behavior to sorption. Paracetamol acted as a negative control because it has no known tendency to interact with materials, diazepam as a reference of absorption and insulin as marker of adsorption only. Paracetamol is a slightly lipophilic drug (logP = 0.91) with a low Van der Waals volume (representing the volume occupied by one single molecule) and is under a non-ionized form at the studied pH. The slight lipophilic properties of paracetamol coupled with the relatively high concentration at which it is administered might explain that to our knowledge no studies have reported a drug loss due to a sorption phenomenon. Diazepam is a highly lipophilic drug (logP = 3.08), with a still relatively low Van der Waals volume yet bigger than paracetamol (242.85 *versus* 138.08 Å^3^). Diazepam solutions were studied at pH = 5.3, at which diazepam was completely under its non-ionized form. Insulin is a peptide, with a much higher Van der Waals volume (3123.51 Å^3^) and positively charged at the pH of injectable forms (pH 6.4). The presence of a positive charge could explain that insulin has a tendency to adsorb to the material’s surface (by a weak charge interaction). But the combination of charge and important steric hindrance is not in favor of its diffusion inside the polymer material.

Since the length of the tested tubes was different from one to another, the straight reading of the loss of concentration of the API did not allow direct comparison. The effect size was therefore calculated with sorption rates expressed as percentage/cm² in order to compare the influence of materials for each drug sorption. The expression of the effect size allowed us to compare how much sorption with alternative tubings was different from sorption with PVC at a given time (T8 in this study). In clinical research, effect sizes are usually interpreted according to Cohen’s rules defined as follow: small (ES = 0.2), medium (ES = 0.5) and large (ES = 0.8: grossly perceptible and therefore large). In this study, several effect sizes were much larger than 0.8 implying certainly relevant differences from a pharmacological point of view.

The static condition (flow rate = 0 mL/h) was studied in order to create a “worst-case” condition, in which contact between drugs and surface will be at its maximum. On the contrary, dynamic conditions at 1 mL/h and 10 mL/h were simulating clinical use situation. For paracetamol and diazepam, the loss of active product ingredient was more important in static than in both dynamic conditions. Variation during infusions of insulin low concentrations and low flowrate has already been observed^28^, and were imputed to an adsorption phenomena. In our case, insulin loss was less for a flow rate of 10 ml/h than for 1 mL/h, this could be explained by the fact that a faster flowrate would induce a faster saturation of the binding sites. Once all the binding sites were occupied an equilibrium state was reached and the concentrations converge to the T0 concentrations. The interaction of insulin with a saturated surface is not known, if no interactions occur the concentrations would be the same as T0, or the potential loss of insulin could be counterbalanced by the possible desorption of API from the saturated surface. Another possible explanation of this phenomenon could be that at a faster flowrate, the contact time between insulin and material was shorter and thus led to a fewer loss due to adsorption. A possible competition between the sorption interactions and flow driven interactions could also explained the flowrate dependent equilibrium. However, PVC/PU tubings did not induce any API loss during static contact with insulin solutions, while a loss of API was noticed during dynamic contact with a flow rate of 1 mL/h. This could possibly be explained by a competition between the phenolic excipients (phenol and metacresol) entering in the composition of Novorapid® and insulin. According to the Van der Waals volume (90.52 Å^3^ and 107.31 Å^3^) and logP (1.67 and 2.18) of phenol and metacresol, the adsorption could possibly be followed by an absorption phenomenon, thus inducing a difference in the sorption kinetics between insulin and excipient. Insulin adsorption appeared to be a fast phenomenon, highlighted by the interaction in dynamic condition. When increasing contact time in static condition, excipients with a different sorption behavior and kinetics could shift the sorption equilibrium, decrease insulin affinity for the tubing surface, comparatively to dynamic conditions. This hypothesis is in good agreement with data reported by Masse *et al*.^3^, who showed a sorption phenomenon involving metacresol and phenol of a Novorapid diluted solution (1 UI/mL) when in contact with PVC tubings. Based on this result, drugs with an adsorption only profile such as therapeutic peptides or monoclonal antibodies should not be tested in static condition, as a dynamic test at a low flow rate appeared to be more suitable. When increasing the flow rate, the percentage of API lost decreased thus concentrations remained close to the initial ones. With a high flow rate, the volume of solution is higher than with a low flow rate, thus the total quantity of API in contact with the material is also increased. This increase could be in favor of a saturation of the tubing surface, decreasing the tendency to adsorption, or could also cause a faster renewal of the solution which gives less time for the molecules to adsorb onto the tubing wall. Similar results to ours have also been reported for diazepam^8^ and insulin^29^ infusion (less drug loss for faster infusion rates), thus limiting the potential clinical impact for the patient.

The physicochemical characterization of each material was performed by assessing the qualitative composition of the surface in contact with the medication by FTIR spectroscopy, and by measuring the charge (estimated by zeta potential) that could interact with non-ionized or ionized drugs.

PVC was chosen for reference material as it is widely used in IV tubings manufacturing due to its very good mechanical properties (transparency, flexibility) and its low cost. As it has already been observed^8,9,12,13,22^, our results show that PVC had a high tendency to absorb diazepam (at 1 mL/h, the loss was comprised between 85.58% and 93.91% of initial concentration) and also induced insulin adsorption (loss of 32.56% to 43.53% of initial concentration at 1 mL/h), but which was however the least loss amongst all alternative materials for insulin.

The PVC/PU tubings appeared to have a high tendency for sorption phenomena. Compared to PVC, PVC/PU had a negative effect size (indicating a significantly higher tendency for sorption) for the three studied drugs at a flowrate of 1 mL/h. Moreover, PVC/PU had the closest to 0 zeta potential of all the studied materials and could be correlated with its higher tendency to absorb diazepam, but not adsorb insulin. As diazepam was under its non-ionized form, a low surface charge could promote sorption phenomenon and on the other hand this slightly negative charge could interact with positively charged molecules such as insulin. As both paracetamol and diazepam were non-ionized in the condition of this study, a low charge surface could have been favorable for interaction between drug and material. However, PU tubings have been shown to behave very differently depending on the nature of the PU. In a recent study, Foinard *et al*.^2^ highlighted that thermoplastic PU were more prone to absorption of diazepam and isosorbide dinitrate than thermosetting PU. The polyurethane used in this study was of thermoplastic nature, and also showed a high tendency to promote diazepam sorption, which is coherent with their results. It is therefore possible that using a thermosetting PU could yield different sorption results, however it would not be able to be used as a coextruding material.

The PVC/PE was not completely inert as it induced a slight loss of diazepam (ranging from 8.95% to 15.25% at 1 mL/h), but interacted much more with insulin (losses ranging from 70.38% to 75.09% at 1 mL/h). Like for PVC/SEBS tubings, PVC/PE tubings presented the most important loss of insulin compared to PVC alone, this observation could be related to zeta potential measurement as PVC/SEBS and PVC presented also the lower zeta potential. Insulin is infused at a pH of 6.1 and at this pH is present in a positively charged form, thus interaction between the positive charge of the drug and the negative charge of the surface could have been promoted. The impact of zeta potential could be more accurately estimated in further studies by assessing the zeta potential as a function of pH. The results presented here can be correlated with previous data already reported by other authors indicating interactions between insulin and PE tubings^14,17,30,31^.

As expected, coextruded SEBS and SEBS were both styrenic thermoplastic elastomers with a very similar composition as shown by FTIR spectroscopy. PVC/SEBS samples had the lowest zeta potential and yet were more prone to absorption of diazepam than PE, SEBS and TPO, as shown by effect size results. Surface charge is not the only factor affecting drug sorption. Based on the FTIR result, it can be hypothesized that TOTM was present in the SEBS analyzed layer. As TOTM was used as a plasticizer in the external PVC layer, it was not supposed to be present in the SEBS layer. The presence of TOTM could have therefore modified the surface properties of the coextruded SEBS and allowed diazepam to absorb more easily. The impact of the plasticizer’s amount in the sorption process has already been shown for PVC by Treleano *et al*.^21^ and by Al Salloum *et al*.^20^, but this is to our knowledge the first published example of its influence on promoting sorption phenomena in other materials. Monolayered PE tubings available on the market were not selected for this study as they are generally not considered to be adequate for infusion medical tubings as their rigidity is too high and they cannot be clamped without altering the tubing. In the field of infusion, manufacturers prefer to associate the PE with PVC in order to maintain the flexibility of tubings, particularly infusion sets. According to its mechanical properties, TPO was therefore chosen as a PVC free alternative. TPO is an olefin thermoplastic elastomer whose exact chemical structure is not publically available. Its behavior was close to that of SEBS and PE, but diazepam sorption was more important in static condition with TPO tubings. Moreover, even if the effect size calculated at the final analytical time gave a higher value than SEBS, the evolution overtime was different and showed a higher loss of diazepam.

In summary, PVC/PE and a thermoplastic elastomer alternative (SEBS) alone or coextruded with PVC presented a better behavior than PVC alone, as absorption was decreased, especially when in contact with diazepam solutions. The loss was less important with these 3 materials even at a high flow rate of 10 mL/h. However, PVC seemed to behave least badly than other studied tubings with regards to insulin adsorption.

Measuring the surface zeta potential was an innovative approach to explain drug sorption phenomenon, and the results obtained in this study are promising but further analysis needs to be performed to assess if materials’ surface charge has a critical influence on sorption phenomenon or not. In order to ensure comparison between materials, the surface zeta potential of the IV tubings was measured only at pH = 5, yet the diluted drug solutions that were administered in a simulated clinical setting were at various pH. This study focused on three drugs, in respect with the recommendation for their administration. In such conditions, the API were positively charged or neutral. No pH adjustments were made in order to assess the phenomenon as it can occur during clinical use. Changing the pH of each drug solution to assess the sorption profiles at extreme pH where the drugs would be charged differently could help better understand the mechanisms involved in the sorption phenomenon, but is experimentally difficult to undertake due to the potential instability of the drugs at these pH. Further studies with negatively charged drugs (like zoledronic acid) would be of interest to determine the impact of surface charge potential in drug sorption. Also, additional analyses of the materials could also be performed at multiple pH magnitudes in order to get a zeta potential profile that will help to evaluate the usefulness of surface zeta potential to estimate the sorption tendency of drugs with materials.

Even though none of the coextruded material completely prevented plasticizer migration, the release of TOTM appeared to be decreased with all coextruded materials compared to PVC tubings. Adding a coextruded inner layer to PVC tubings can decrease TOTM migration^19^ and reduce absorption of small drugs, which is also what we confirmed but with results varying with the nature of the coextruded material. Amongst all coextruded alternatives, PVC/PU tubings was the one with the least protective impact on TOTM release. The data presented in our study is in favor of the presence of TOTM at the surface of the SEBS coextruded layer, possibly caused by either a migration from the PVC matrix or by surface contamination during the manufacturing process, but further studies throughout the whole SEBS layer need to be performed to be able confirm or not these hypotheses. As SEBS is a styrenic based block copolymer, the aromatic ring in the styrene function could present an affinity for TOTM which also possesses an aromatic ring. The presence of TOTM between the SEBS polymer chains could have modified the matrix structure and have potentially promoted drug sorption into the coextruded inner layer. Based on this result, non-coextruded thermoplastic elastomers appear as an interesting alternative for the manufacturing of infusion tubings as they could combine a limited tendency to promote drug sorption and would be plasticizer free, limiting the potential clinical impact for the patient of both content-container interaction (sorption and release). However, leachables and extractibles originating from the elastomers were not assessed in this study and should be evaluated in order to perform a complete characterization of the material.

This study was performed with commercial medications, following recommendations for medical devices use and drugs reconstitution at clinical used concentrations. Yet, a high concentration could have masked slight variation of API concentration. Commercial medications are composed of API and excipients which are diluted or not in a dilution solvent (0.9% NaCl or 5% glucose). This study has shown the variation of API concentration but did not assess the potential variation of excipient concentration or impact of dilution solvent. Excipient could have been in competition with API leading to an underestimation of the loss or on the opposite could have promoted API sorption.

## Conclusion

Sorption is a complex process involving several parameters of the material and the drug at the same time making it very complex to predict. None of the studied materials was inert with all drugs but SEBS and TPO along with PVC/PE appeared to induce less absorption phenomena and thus represented very interesting alternatives to PVC tubings. Moreover, the use of PVC based coextruded alternatives also decreased the ability of plasticizer to migrate from the PVC matrix migration, especially for PVC coextruded with PE.

The measure of the zeta potential appeared to be an interesting tool to characterize the inner surface of the tubings by highlighting that differences in zeta potential could be related to different sorption behavior. Further studies will also be necessary to precise the impact of plasticizer migration in the coextruded layer upon sorption phenomena.

## Supplementary information

Supplementary data

## Data Availability

The data that support the findings of this study are available on request from the corresponding author, P. Chennell.
