# Supplementary material for: Impact of alternative materials to plasticized PVC infusion tubings on drug sorption and plasticizer release
Source: Sci Rep. 2019 Dec 12;9:18917. doi: 10.1038/s41598-019-55113-x (PMC6908714; doi:10.1038/s41598-019-55113-x)
Supplement: Supplementary file 1 — Supplementary data [file 41598_2019_55113_MOESM1_ESM.pdf]

Impact of alternative materials to plasticized PVC infusion tubings on drug sorption and plasticizer release

N. TOKHADZE[1], P. CHENNELL[1*], L. BERNARD[1], C. LAMBERT[2], B. PEREIRA[2], B. MAILHOT-JENSEN[3], V. SAUTOU[1]

[1] UNIVERSITE CLERMONT AUVERGNE, CHU CLERMONT FERRAND, CNRS, SIGMA CLERMONT, ICCF, F-63000 CLERMONT-FERRAND, France

[2] UNITE DE BIOSTATISTIQUES (DELEGATION A LA RECHERCHE CLINIQUE ET A L'INNOVATION), CHU DE CLERMONT-FERRAND, 63000 CLERMONT-FERRAND, France

[3] UNIVERSITE CLERMONT AUVERGNE, CNRS, SIGMA CLERMONT, ICCF, F-63000 CLERMONT-FERRAND, France

Supplementary data

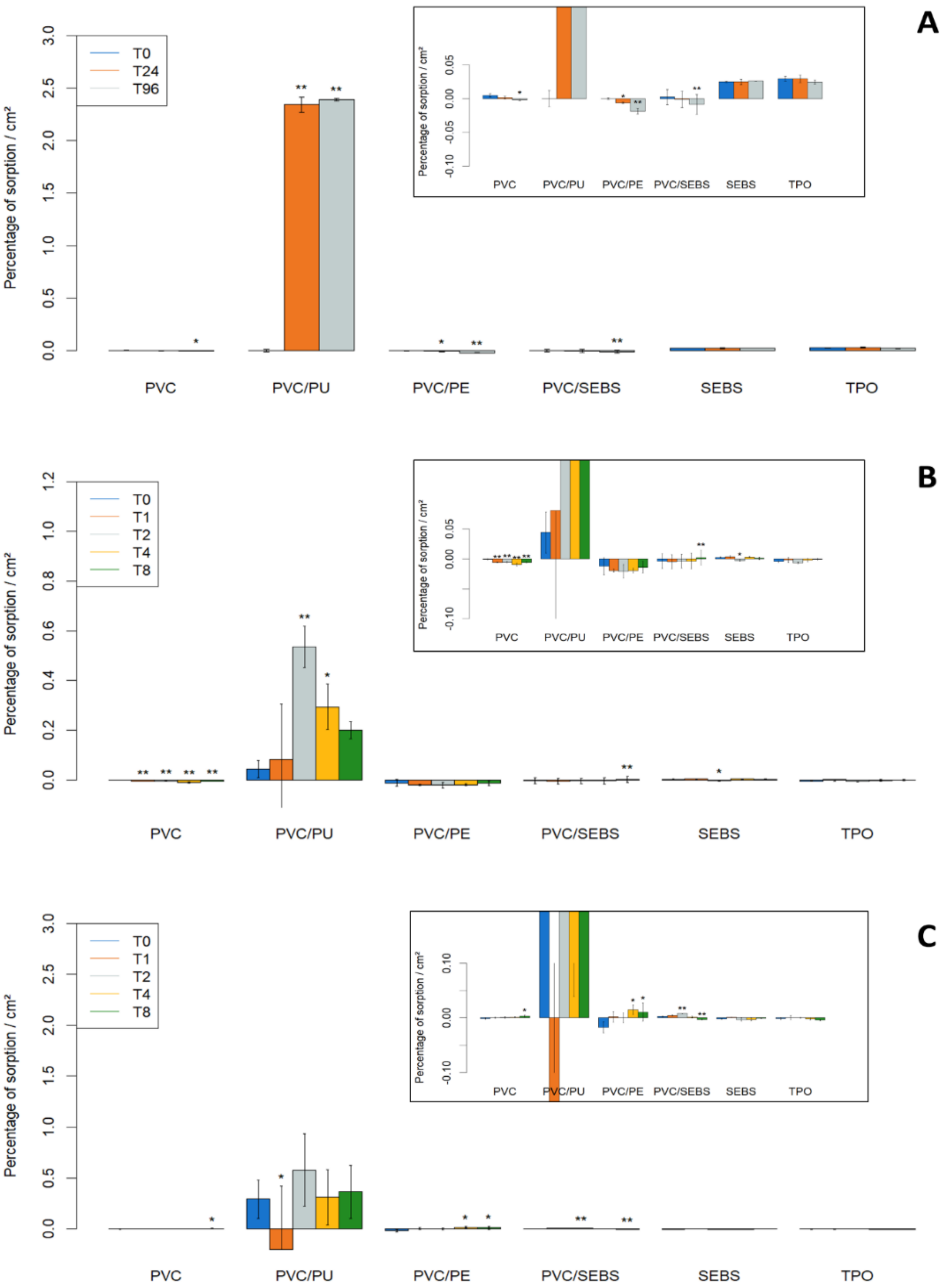

Figure A: Paracetamol sorption/cm² for each IV tubings in static condition (A), 1mL/h (B) and 10 mL/h (C) dynamic conditions. (n=3, mean ± standard error of mean; p<0.05: *; p<0.001: **)

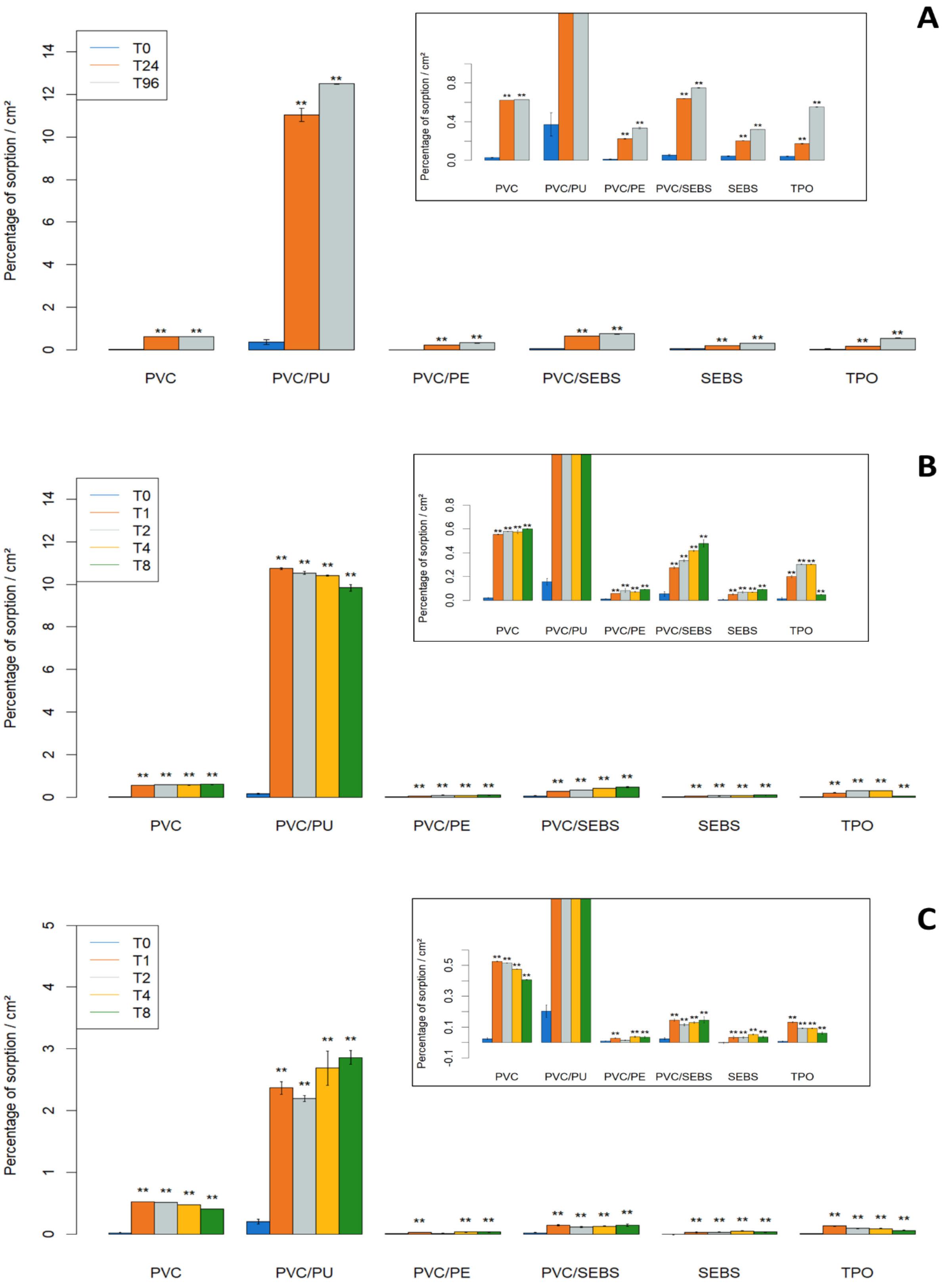

Figure B: Diazepam sorption/cm² for each IV tubings in static condition (A), 1mL/h (B) and 10 mL/h (C) dynamic conditions. (n=3, mean ± standard error of mean; p<0.05: *; p<0.001: **)

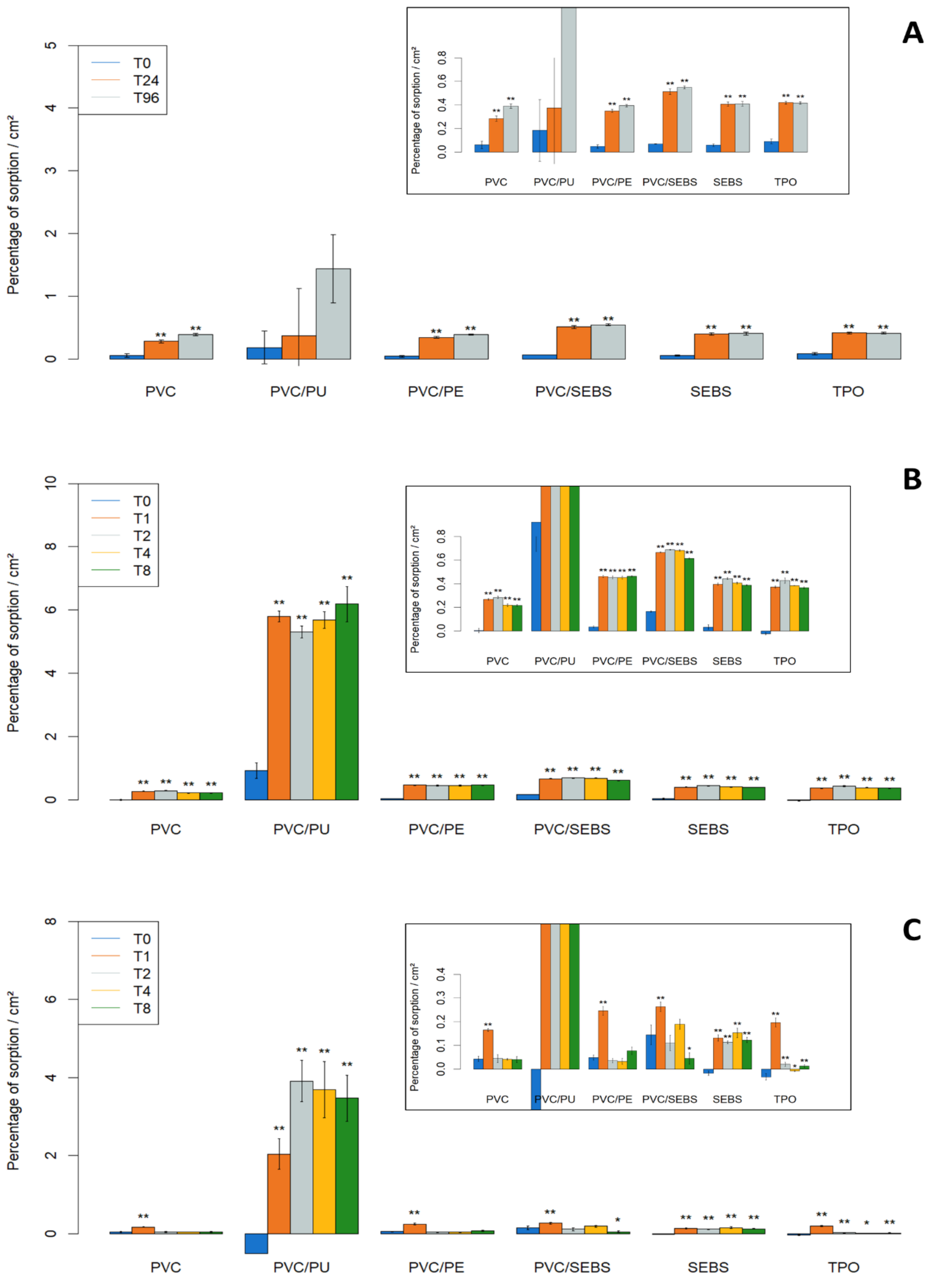

Figure C: Insulin sorption/cm² for each IV tubings in static condition (A), 1mL/h (B) and 10 mL/h (C) dynamic conditions. (n=3, mean ± standard error of mean; $p<0.05$: *; $p<0.001$: **)

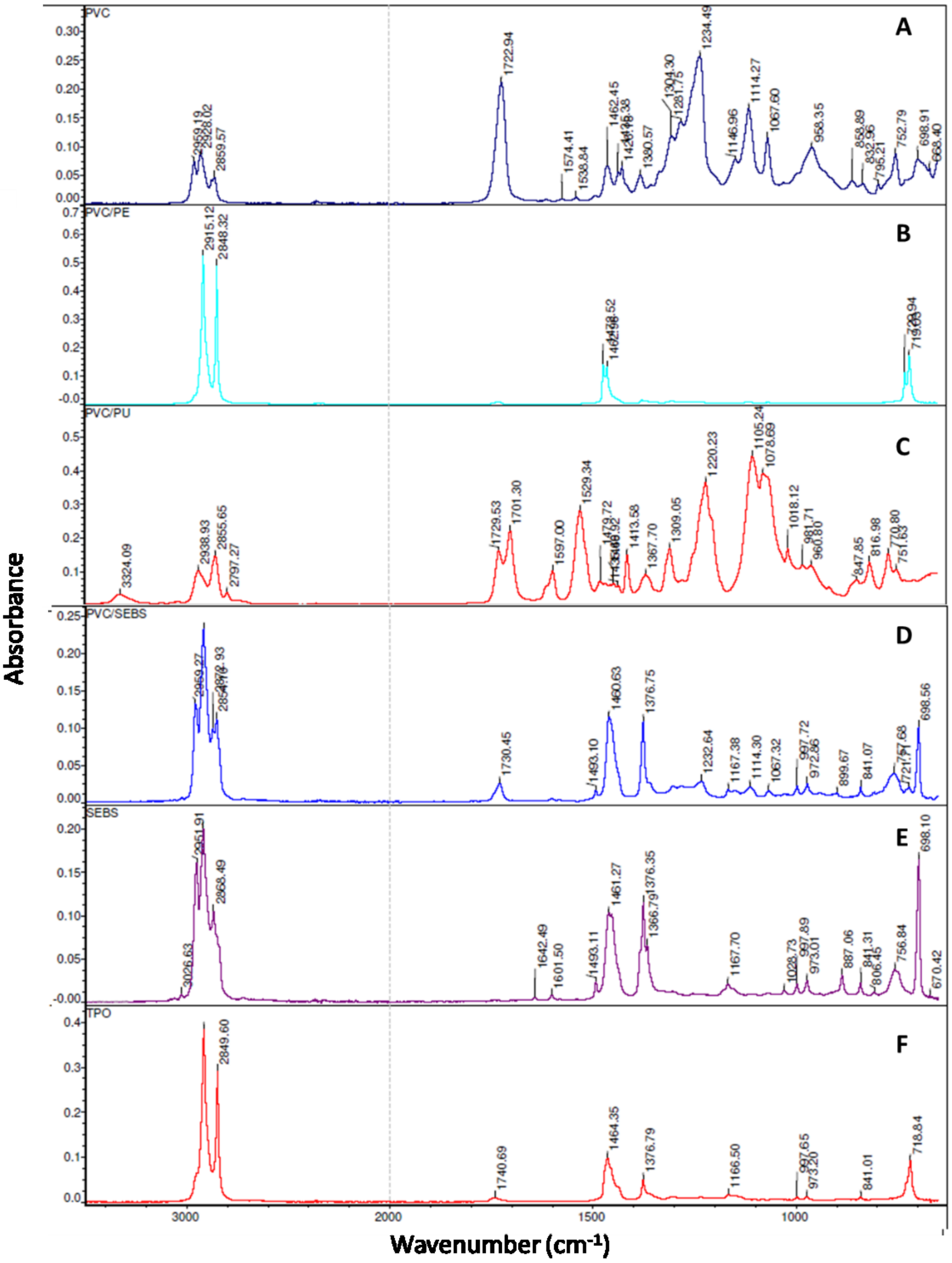

Figure D : Inner surface of each tubing analyzed by FTIR spectroscopy : PVC (A), PVC/PE (B), PVC/PU (C), PVC/SEBS (D), SEBS (E), TPO (F).
